# Nitrogen Immobilization by Wood Fiber Substrates Strongly Affects the Photosynthetic Performance of Lettuce

**DOI:** 10.3390/plants14101518

**Published:** 2025-05-19

**Authors:** Lingyi Wu, Ruohan Li, Juncheng Liu, Wenzhong Cui, Zhiyong Qi, Wanlai Zhou

**Affiliations:** 1 Institute of Urban Agriculture, Chinese Academy of Agricultural Sciences, Chengdu 610213, China; 82101225393@caas.cn (L.W.); 82101235419@caas.cn (R.L.); 2 School of Mechanical Engineering, Chengdu University, Chengdu 610100, China; ljceng@icloud.com (J.L.); 17784240198@163.com (W.C.)

**Keywords:** chlorophyll fluorescence, JIP test, PSI, PSII

## Abstract

Wood fiber substrates are widely used as peat substitutes in horticulture, but the impact of their high carbon-to-nitrogen ratio on nitrogen immobilization and crop photosynthetic performance remains unclear. This study systematically examined the effects of wood fiber substrates on lettuce photosynthetic performance and underlying physiological mechanisms using pot experiments. Two substrate treatments—peat (control) and wood fiber—were combined with three nitrogen levels: low, medium, and high (63, 127, and 210 mg N·L^−1^). Results indicated that wood fiber substrates significantly reduced the availability of fast-acting nitrogen, leading to a substantial decrease in lettuce biomass (39.0–56.8%), total nitrogen content (7.2–39.9%), and chlorophyll content (13.7–36.2%). Chlorophyll fluorescence kinetics analysis revealed that wood fiber substrates impair photosystem function through multiple pathways. At the early stage (15 days), key effects included structural damage to the donor side of PSII(Photosystem II), indicated by L and K peaks, and inhibited electron transfer on the PSI(Photosystem I) acceptor side (δRo decreased by 15.08–27.90%, along with a reduction in W_OI_ amplitude). The findings provide an important theoretical basis for optimising nitrogen management strategies for wood fibre substrates.

## 1. Introduction

Peat, the most widely used substrate [1,2], is non-renewable and poses significant ecological concerns [3,4]. To address this issue, the horticultural industry is actively seeking sustainable alternative materials [5,6,7]. Among these alternatives, wood fiber has emerged as a promising peat substitute due to its unique physical properties—low bulk density, high porosity, and good rewettability—as well as its eco-friendliness [8,9].

It is now gaining widespread global adoption. Despite its advantages in soilless cultivation, the high carbon-to-nitrogen (C/N) ratio of wood fiber can induce nitrogen immobilization, which may negatively affect plant growth [10]. Nitrogen immobilization of the cultivation substrate refers to the phenomenon in which inorganic nitrogen from fertiliser sources is fixed in the substrate due to the unique physicochemical properties of the substrate’s organic materials and is difficult to be absorbed and utilised by the crop [3,11,12]. Because the exogenous nitrogen is partially immobilised by the substrate material, the amount of nitrogen that the plant can take up becomes less. Studies have shown that microbial nitrogen immobilization significantly increases when the substrate’s C/N ratio exceeds 30:1, thereby reducing plant-available nitrogen [13,14,15]. However, wood fiber substrates typically exhibit C/N ratios of 100:1 or higher, making nitrogen immobilization a significant concern [16]. Numerous studies have shown that Woody Substrate (WS), such as wood fibre, wood chip substrate, bark substrate, etc., tends to have severe nitrogen fixation during crop cultivation, leading to crop nitrogen deficiency, which greatly increases the difficulty of nitrogen and nutrient management in WS cultivation [17,18]. Gruda et al. [19] reported that complete wood fiber substrates exhibited significant nitrogen immobilization, leading to tomato growth inhibition. Wright et al. [12] found that plants grown in wood-fractionated substrates required an additional 100 mg·L^−^^1^ of nitrogen to achieve growth comparable to peat-based cultivation. These findings indicate that nitrogen immobilization in wood fiber substrates may severely restrict their horticultural applications, highlighting the urgent need for optimization through scientific nitrogen management strategies.

Nitrogen availability plays a crucial role in shaping the structure and function of the photosynthetic system, while nitrogen deficiency severely disrupts the stability and efficiency of the photosynthetic apparatus [20]. Chlorophyll a fluorescence measurement is a widely used technique for evaluating the impact of stress factors on photosynthesis [21]. The JIP test, a robust mathematical model developed by Strasser et al. [22], quantifies energy uptake by the PSII antenna complex, energy capture in the PSII reaction center, electron transport from PSII to PSI, and the final reduction of the PSI electron acceptor. The JIP test has been widely employed to assess crop responses to nitrogen deficiency [23,24,25]. Nitrogen deficiency decreases chlorophyll a and accessory pigments while altering the structure of chloroplast-like vesicle membranes [23]. This typically results in impaired PSII reaction center function, a reduced pool of electron acceptors, disrupted electron transfer processes, and diminished photochemical efficiency [20,24].

Nitrogen immobilization in woody substrates reduces plant-available nitrogen, thereby significantly impacting plant growth and physiological functions. While numerous studies have explored the effects of nitrogen deficiency on plant photosynthesis, research on plant photosynthetic responses to nitrogen immobilization in wood substrates remains limited. Moreover, whether nitrogen deficiency caused by immobilization in wood-fiber substrates induces physiological effects similar to low-nitrogen stress lacks systematic investigation.

Based on this, this study systematically examined how nitrogen immobilization in wood fiber substrates affects lettuce photosynthesis and its underlying mechanisms. Potting experiments were conducted with two substrate types (peat and wood fiber) and three nitrogen levels (low, medium, and high). Chlorophyll fluorescence kinetics and photosynthetic parameters were analyzed to elucidate these effects. In this study, it was hypothesised that nitrogen fixation by wood fibre substrates would have a negative effect on the photosynthetic performance of lettuce. The findings will contribute to optimizing nitrogen management strategies for wood fiber substrates and facilitating their efficient use in soilless cultivation.

## 2. Results

### 2.1. Fast-Acting Nitrogen Supply in the Substrate

**Figure 1:**
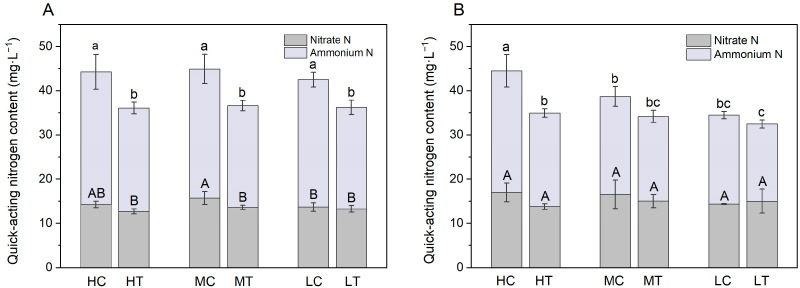
Concentration of quick nitrogen in the substrate under different treatments ((**A**) is for Day 15; (**B**) is for Day 30). High nitrogen & control substrate (HC), high nitrogen & wood fiber substrate (HT), medium nitrogen & control substrate (MC), medium nitrogen & wood fiber substrate (MT), low nitrogen & control substrate (LC), and low nitrogen & wood fiber substrate (LT). Within each organic acid or germination index, values that do not share a letter are significantly different according to ANOVA and Duncan’s multiple range test (*p* < 0.05).

**Figure 2:**
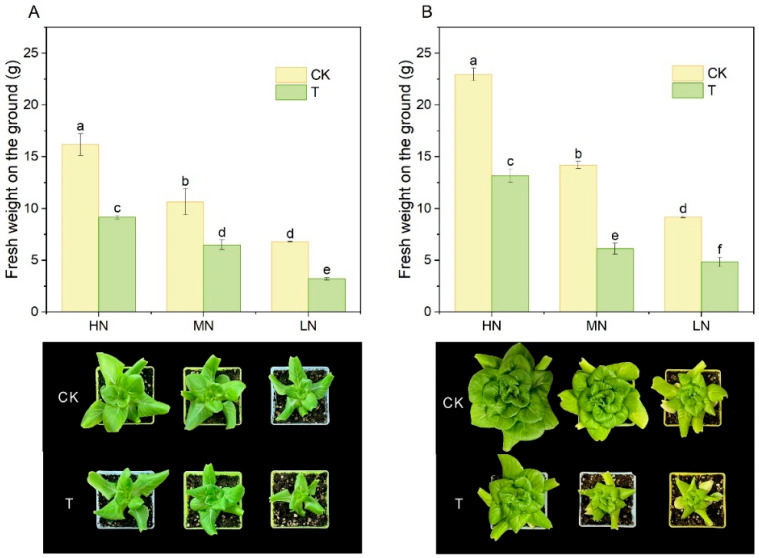
Above ground fresh weight and morphological characteristics of plants under different treatments ((**A**) for Day 15 and (**B**) for Day 30). High nitrogen (100% N, HN), medium nitrogen (60% N, MN), and low nitrogen (30% N, LN). Control substrate (CK) and wood fiber substrate (T). Within each organic acid or germination index, values that do not share a letter are significantly different according to ANOVA and Duncan’s multiple range test (*p* < 0.05).

N immobilization by wood fiber substantially reduced fast-acting nitrogen supply in the substrate (Figure 1). At day 15, the ammonia nitrogen content in the wood fiber substrate was reduced by 22.0%, 21.2% and 20.2% compared to the control at high, medium and low nitrogen application levels, respectively, with all differences being statistically significant (*p* < 0.05); At day 30, that reductions were 23.1%, 13.4% and 13.3%, respectively. Nutrient measurements were taken 2–3 days after applying the nutrient solution, reflecting the residual nitrogen state in the substrate following both lettuce uptake and nitrogen immobilization by the substrate. Although the total nitrogen applied to the wood fiber substrate was equivalent to that of the control substrate, the biomass and total nitrogen content of the lettuce grown in it were significantly lower, suggesting reduced nitrogen uptake by plants in the wood fiber substrate (Figure 2). Theoretically, the remaining quick nitrogen concentration in the wood fiber substrate should be higher than that in the control; however, actual measurements revealed a significantly lower concentration of quick nitrogen. This paradox suggests that the wood fiber substrate significantly reduces the availability of quick-acting nitrogen by strongly fixing nitrogen and converting exogenous inorganic nitrogen into organic forms, which are less accessible to plants. Table 1 shows that the wood fiber substrate has a high nitrogen immobilization potential, reaching 115 mg·L^−1^ within 4 days, equivalent to 18.40 mg per pot of substrate. Over the 30-day experimental period, the total N input per tray of substrate was approximately 67.30 mg, 40.32 mg and 20.00 mg for high, medium and low N applications, respectively, whereas wood fiber fixes between 27.3% and 92.0% of the applied N in 4 days, which is sufficient to result in a significant reduction in the supply of fast-acting N to the substrate.

### 2.2. Lettuce Growth Performance and Nutrient Accumulation

Wood fiber appeared to have a significant negative effect on the growth performance of lettuce (Figure 2).The aboveground fresh weight of lettuce in the wood fiber substrate was reduced by 43.5%, 39.0% and 52.9% at day 15 compared to the control at high, medium and low nitrogen application levels, respectively; at day 30, this reduction was 42.6%, 56.8% and 47.2%, with the difference reaching the level of significance on both occasions (*p* < 0.05). Morphological characteristics of lettuce also showed that at the same level of nitrogen application, the control plants had larger and thicker smooth leaves with intense green colour, while the wood fiber group had shorter plants and narrower leaves. By day 30, this morphological difference was even more pronounced, with wood fiber-based lettuce showing significant nitrogen deficiency characteristics such as short plants and yellowed leaf ends.

**Figure 3:**
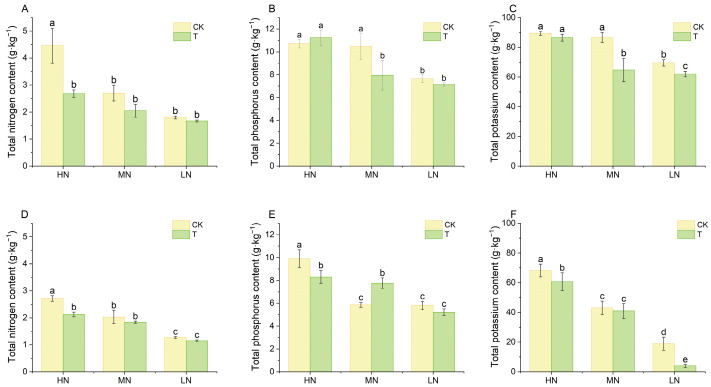
N, P, and K contents of plants under different treatments ((**A**–**C**) for Day 15 and (**D**,**F**) for Day 30). High nitrogen (100% N, HN), medium nitrogen (60% N, MN), and low nitrogen (30% N, LN). Control substrate (CK) and wood fiber substrate (T). Within each organic acid or germination index, values that do not share a letter are significantly different according to ANOVA and Duncan’s multiple range test (*p* < 0.05).

Further, nutrient accumulation in lettuce cultivated on wood fiber substrate was also significantly lower than the control (Figure 3). Nitrogen content in lettuce gradually decreased with decreasing nitrogen application, both in wood fiber substrate and control, indicating that nitrogen availability directly affected nitrogen accumulation in lettuce leaves under the conditions of this experiment. The N content of lettuce in the wood fiber substrate was reduced by 39.9%, 24.2%, and 7.2% at day 15 compared to the control at high, medium, and low N application levels; at day 30, this reduction was 21.7%, 9.5%, and 9.9%, respectively. The accumulation of phosphorus and potassium in leaves of lettuce cultivated on wood fiber substrates at different levels of nitrogen application showed a similar pattern to that of nitrogen, being significantly lower than that of the control substrate in most cases, and decreasing with decreasing nitrogen application.

### 2.3. Chlorophyll a Fluorescence Properties

#### 2.3.1. OJIP Chlorophyll Fluorescence Kinetics

**Figure 4:**
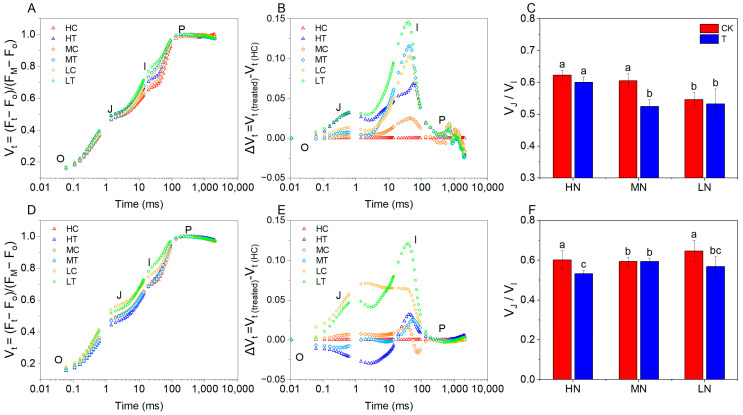
Relative variable fluorescence curves and V_J_/V_I_ of lettuce under different treatments ((**A**–**C**) for Day 15 and (**C**–**F**) for Day 30). High nitrogen (100% N, HN), medium nitrogen (60% N, MN), and low nitrogen (30% N, LN). Control substrate (CK) and wood fiber substrate (T). High nitrogen & control substrate (HC), high nitrogen & wood fiber substrate (HT), medium nitrogen & control substrate (MC), medium nitrogen & wood fiber substrate (MT), low nitrogen & control substrate (LC), and low nitrogen & wood fiber substrate (LT). Within each organic acid or germination index, values that do not share a letter are significantly different according to ANOVA and Duncan’s multiple range test (*p* < 0.05).

The OJIP chlorophyll fluorescence kinetics can well reflect the changes in primary photochemical reactions and photosynthetic functions of PSII [26]. OJIP is the four key stages of the Chlorophyll Fluorescence Induction Kinetics (CFIK) curve for studying the functional state of photosynthetic system II (PSII).It reflects the efficiency of light energy absorption, transfer and electron transfer in PSII by irradiating dark-adapted plant leaves with high-intensity light and recording changes in fluorescence intensity on millisecond time scales. The chlorophyll fluorescence induction curves for all treatments showed a typical OJIP shape (Figure 4A,D). To visualise the comparative effects of the treatments on transient kinetics, the curves were plotted as relative variable fluorescence V_t_ = (F_t_ − F_o_)/(F_M_ − F_o_) and ΔV_t_ = F_t(treated)_ − F_t(HC)_ (Figure 4B,E). Rising O-J step in the OJIP curve correlates with the degree of closure of the PSII reaction center [24]. The J-I part of the curve corresponds to the reduction of the secondary electron acceptor Q_B_, plastoquinone (PQ), cytochrome (Cyt b6f), and plastocyanin (PC) [21]. Fluorescence transient analysis showed that the V_t_ of lettuce under different treatments showed significant divergence at points J and I, especially at point I, indicating that the effect of wood fiber substrate on lettuce mainly occurred at the O-J and J-I periods.

To further explore the initial site of action of nitrogen nutrition, the JIP test parameter V_J_/V_I_ was introduced (Figure 4C,F). At 15 days of age, V_J_ and V_I_ increased with decreasing nitrogen application in both wood fiber and control. Whereas, at the same level of nitrogen application, both V_J_ and V_I_ were significantly higher in the wood fiber group than in the control group. Considering that the wood fiber substrate itself reduces the effective supply of nitrogen, the above facts suggest that reduced nitrogen supply can lead to elevated V_J_ and V_I_, reflecting the openness of the PSII reaction center in lettuce and reduced electron transfer from Q_A_^−^ to Q_B_^−^ on the PSII receptor side. It is noteworthy that only the wood fiber group had significantly higher V_J_ and V_I_ at both low and medium N levels, whereas only V_I_ was significantly higher in the other treatments, suggesting that the effect of nitrogen deficiency firstly affected V_I_, and only when nitrogen deficiency was severe to a certain extent did it further lead to a significant increase in V_J_. At 30 days of age, V_J_ and V_I_ showed a significant increase in V_J_ and V_I_ in and only in the low nitrogen treatments, and there was no significant difference between wood fiber and control, suggesting that the effect of the wood fiber substrate was weakening with time, but that the effect of severe low nitrogen continued to affect the chlorophyll fluorescence performance of lettuce. In addition, at the same level of nitrogen application, both V_J_/V_I_ were lower in the wood fiber group than in the control group, suggesting that nitrogen immobilization by wood fiber had a greater effect on the lettuce I step.

**Figure 5:**
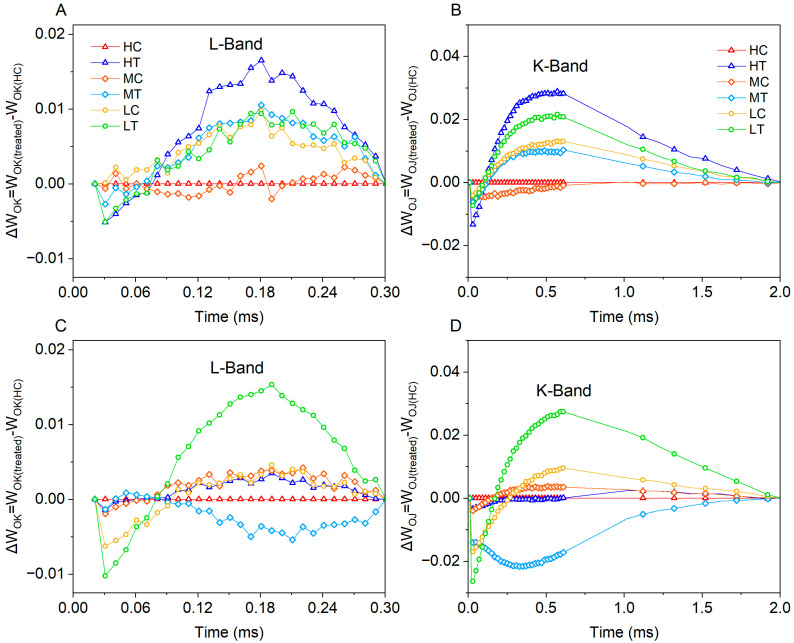
ΔW_OK_, ΔW_OJ_ of lettuce under different treatments ((**A**,**B**) are for Day 15, (**C**,**D**) are for Day 30). High nitrogen & control substrate (HC), high nitrogen & wood fiber substrate (HT), medium nitrogen & control substrate (MC), medium nitrogen & wood fiber substrate (MT), low nitrogen & control substrate (LC), and low nitrogen & wood fiber substrate (LT).

To further assess the events reflected in the OK, OJ, OI and IP phases, additional normalisations and corresponding subtractions (differential kinetics) of the fluorescence rise kinetic curves are also presented. Fluorescence rise kinetic curves for the different treatments were double normalised between O (20 μs) and K steps (300 μs) to show the L peak, and differential kinetics were plotted on a linear time scale from 0 to 300 μs, W_OK_ = (F_t_ − F_O_)/(F_K_ − F_O_), ΔW_OK_ = W_OK(treated)_ − W_OK(HC)_ (Figure 5A,C). Simultaneous double normalisation between O (20 μs) and J-steps (2 ms) was performed to show the K-peak and to plot the differential dynamics on a linear time scale from 0 to 2 ms, W_OJ_ = (F_t_ − F_O_)/(F_J_ − F_O_), ΔW_OJ_ = W_OJ(treated)_ − W_OJ(HC)_ (Figure 5B,D). At 15 days of age, lettuce in the wood fiber group showed distinct L and K peaks at all levels of nitrogen application, whereas the control group showed L and K peaks only at low nitrogen. Surprisingly, the wood fiber group exhibited the highest L and K peaks at high nitrogen levels, which usually implies the most intense adversity stress. at 30 days of age, significant L and K peaks were only evident in low-nitrogen wood fiber lettuce.

**Figure 6:**
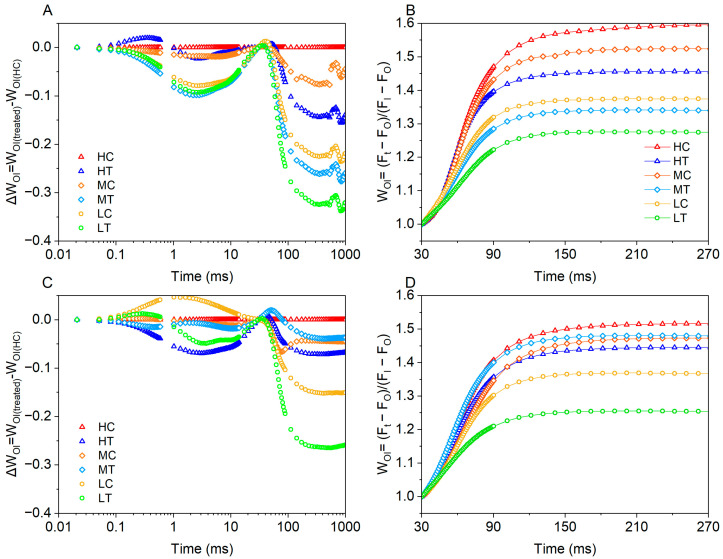
W_OI_, ΔW_OI_ of lettuce under different treatments ((**A**,**B**) are for Day 15, (**C**,**D**) are for Day 30). High nitrogen & control substrate (HC), high nitrogen & wood fiber substrate (HT), medium nitrogen & control substrate (MC), medium nitrogen & wood fiber substrate (MT), low nitrogen & control substrate (LC), and low nitrogen & wood fiber substrate (LT).

As shown in Figure 6, the chlorophyll fluorescence transients of each group were double normalised between F_O_ and F_I_ and expressed as relative variable fluorescence W_OI_ = (Ft − Fo)/(F_I_ − Fo), ΔW_OI_ = W_OI(treated)_ − W_OI(HC)_, F_I_ = Ft = 30 ms, and plotted W_OI_ ≥ 1 over the 30–270 ms time range to show how the IP phase was affected. ΔW_OI_ was used to show the effect of different treatments on the J-step, and the results were generally consistent with ΔVt. The amplitude of the I-P phase in the W_OI_ ≥ 1 fraction reflects the size of the terminal electron acceptor pool on the receptor side of photosystem I (PSI), with smaller amplitudes indicating a smaller terminal electron acceptor pool on the PSI receptor side [24]. The wood fiber substrate as well as the amount of nitrogen applied affected the amplitude of the W_OI_ curves to varying degrees. At 15 days of age, the amplitude of the W_OI_ curve decreased with decreasing nitrogen application, both for wood fiber substrate and control. At the same level of nitrogen application, the W_OI_ amplitude of lettuce in the wood fiber group was even lower. The W_OI_ performance of the different treatments at 30 days of age was essentially the same as at 15 days of age. Considering that the wood fiber substrate itself reduces the effective supply of nitrogen, the above facts suggest that the reduction in nitrogen supply reduces the terminal electron receptor pool on the PSI receptor side and that this inhibition persists over the 30-day cultivation period.

#### 2.3.2. Photosynthetic Electron Transfer

**Figure 7:**
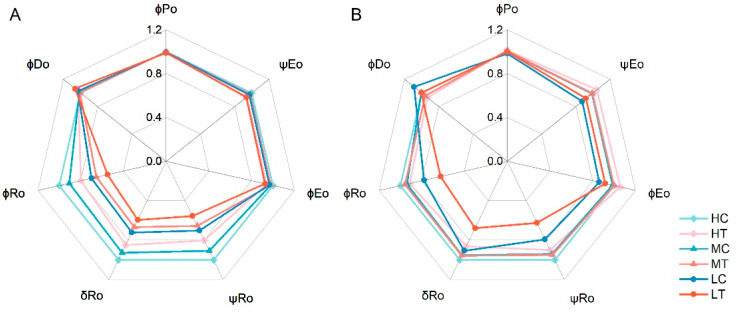
Normalised spider plots of quantum yield of PSII-active reaction centers in lettuce under different treatments ((**A**) for Day 15 and (**B**) for Day 30). High nitrogen & control substrate (HC), high nitrogen & wood fiber substrate (HT), medium nitrogen & control substrate (MC), medium nitrogen & wood fiber substrate (MT), low nitrogen & control substrate (LC), and low nitrogen & wood fiber substrate (LT).

The quantum yield-related data were normalized and visualized as a spider plot to illustrate changes in lettuce quantum yield, from photon uptake to the electron acceptor at the PSI terminal, under each treatment. As shown in Figure 7, at 15 days, the maximum quantum yield for primary photochemistry (φPo), the probability of electron transfer beyond Q_A_^−^ (ψEo), and the quantum yield for electron transport (φEo) did not differ significantly. Subsequently, electron transfer efficiency diverged significantly. δRo represents the probability of electron transport from the reduced intersystem electron acceptor (RE) to the final electron acceptor in PSI. At 15 days, δRo was significantly lower in the wood fiber group than in the CK under the same nitrogen level. The reductions were 15.08%, 27.90%, and 17.68% at high, medium, and low nitrogen levels, respectively. Similarly, φRo, the quantum yield for the reduction of end electron acceptors at the PSI acceptor side (RE), decreased by 20.22%, 27.84%, and 21.46%, respectively. At 30 days, similar to ΔV_t_ trends, differences in electron transfer efficiency diminished across treatments, except for the two low-nitrogen treatments (LC, LT).

**Figure A1:**
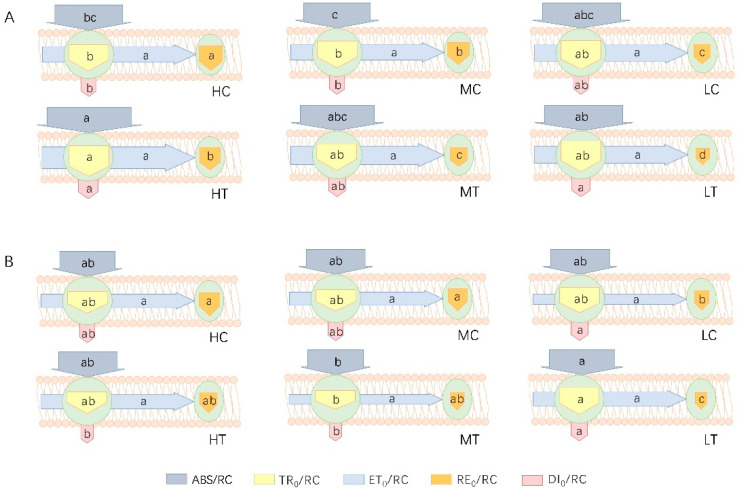
Membrane-specific flux modelling of Energy pipelines in lettuce under different treatments ((**A**), day 15, (**B**), day 30).

Changes in electron transfer energy fluxes at the core of the active reaction were further visualized using an energy pipeline membrane model of specific fluxes in the photosynthetic apparatus (Appendix A Figure A1). The arrow sizes of ABS/RC, TR_0_/RC, ET_0_/RC, RE_0_/RC, and DI_0_/RC represent the efficiencies of light absorption, trapping, electron transport, reduction of electron acceptors, and dissipation, respectively, for each active reaction center (RC). In this study, RE_0_/RC exhibited the most pronounced response to treatments, showing a significant decline in the wood fiber group compared to the control at the same nitrogen level, indicating reduced electron flux from Q_A_^^−^^ to the PSI final electron acceptor. These findings indicate that nitrogen availability significantly influences electron transfer in the lettuce photosynthetic apparatus, with the strongest impact occurring between the reduced intersystem electron acceptor (RE) and the PSI final electron acceptor.

**Figure 8:**
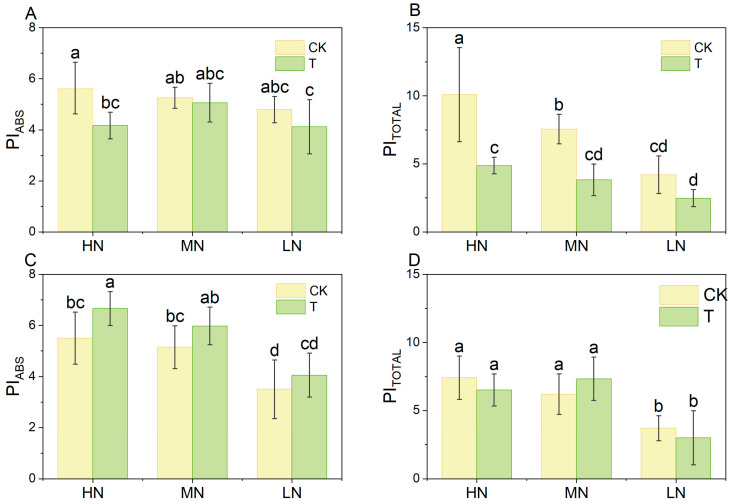
PI_abs_, PI_total_ of lettuce under different treatments ((**A**,**B**) for Day 15 and (**C**,**D**) for Day 30). High nitrogen (100% N, HN), medium nitrogen (60% N, MN), and low nitrogen (30% N, LN). Control substrate (CK) and wood fiber substrate (T). Within each organic acid or germination index, values that do not share a letter are significantly different according to ANOVA and Duncan’s multiple range test (*p* < 0.05).

The performance index PI_abs_ quantifies PSII performance by measuring the efficiency from photon absorption to electron transport chain reduction. PI_total_ represents the efficiency of electron transfer from photon absorption to the PSI terminal electron acceptor, reflecting its performance [27]. As shown in Figure 8, at 15 days, PI_abs_ and PI_total_ were lower in the wood fiber group than in the control (C) group under the same nitrogen level. The reduction in PI_total_ was significantly greater than that in PI_abs_, reaching statistical significance. PI_abs_ decreased by 26.02%, 3.76%, and 13.90%, while PI_total_ decreased by 51.67%, 49.35%, and 40.94% at high, medium, and low nitrogen levels, respectively. This suggests that both PSII and PSI functions were impaired in the wood fiber group, with a more pronounced reduction in the PSI terminal electron acceptor. These findings align with the aforementioned quantum yield and energy flow results. Interestingly, by 30 days, the differences between the wood fiber and control groups had disappeared, suggesting that the nitrogen immobilization-induced damage to the lettuce photosystem may be reversible.

### 2.4. Lettuce Chlorophyll Content and Gas Exchange Parameters

**Figure 9:**
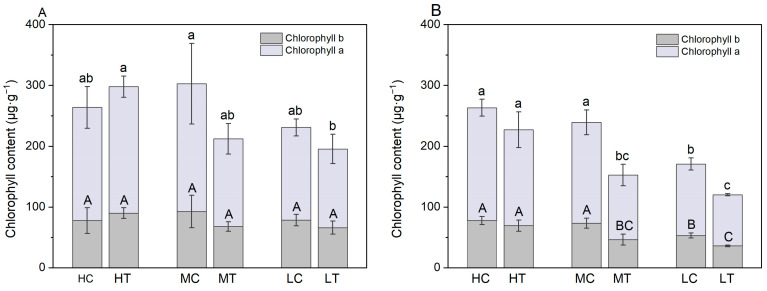
Chlorophyll content of lettuce under different treatments ((**A**) for Day 15 and (**B**) for Day 30). High nitrogen & control substrate (HC), high nitrogen & wood fiber substrate (HT), medium nitrogen & control substrate (MC), medium nitrogen & wood fiber substrate (MT), low nitrogen & control substrate (LC), and low nitrogen & wood fiber substrate (LT). Within each organic acid or germination index, values that do not share a letter are significantly different according to ANOVA and Duncan’s multiple range test (*p* < 0.05).

The chlorophyll content of lettuce cultivated on wood fiber substrate was significantly lower compared to the control (Figure 9). This was particularly evident at 30 days, where chlorophyll a and b contents of wood fiber substrate lettuce were significantly lower (*p* < 0.05) than the control at both medium and low nitrogen levels, and the total chlorophyll content decreased by 13.7%, 36.2%, and 29.6%, respectively, and with decreasing nitrogen application.

**Figure 10:**
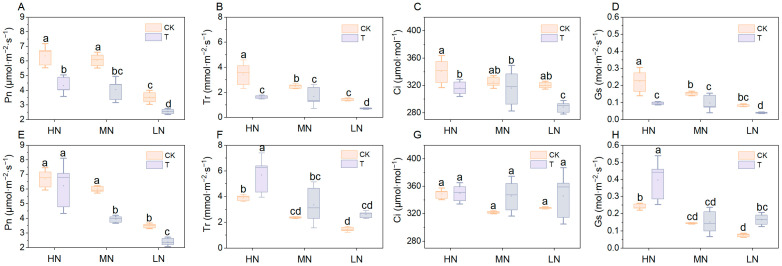
Photosynthetic parameters of lettuce under different treatments ((**A**–**D**) for Day 15 and (**E**–**H**) for Day 30). High nitrogen (100% N, HN), medium nitrogen (60% N, MN), and low nitrogen (30% N, LN). Control substrate (CK) and wood fiber substrate (T). Within each organic acid or germination index, values that do not share a letter are significantly different according to ANOVA and Duncan’s multiple range test (*p* < 0.05).

Wood fiber substrates significantly reduced the net photosynthetic rate of lettuce (Figure 10). The net photosynthetic rate of lettuce gradually decreased as nitrogen levels decreased. At the same nitrogen level, the net photosynthetic rate of lettuce with wood fiber substrates was lower than that of the control. At 15 days, the net photosynthetic rate of the wood fiber group was significantly lower than that of the control group at high, medium, and low nitrogen levels, with decreases of 32.1%, 33.2%, and 27.5%, respectively (*p* < 0.05). At 30 days, the decreases were 7.2%, 34.1%, and 31.3%, respectively, and were significant (*p* < 0.05) at medium and low nitrogen levels. Interestingly, stomatal conductance, intercellular CO_2_ concentration, and transpiration rate were lower in the wood fiber group compared to the control group at the same nitrogen level. However, at 30 days, these parameters were higher in the wood fiber group, indicating that CO_2_ was not efficiently utilized after entering the leaves, leading to Ci accumulation. This suggests that at 15 days, photosynthesis in the wood fiber group is primarily limited by stomatal factors, while at 30 days, it is limited by non-stomatal factors [28]. This difference supports the idea that the mechanism of photosynthesis inhibition by wood fiber substrates exhibits significant stage-dependent characteristics.

## 3. Discussion

### 3.1. Effect of Wood Fiber Substrate on Nutrient Supply and Growth and Development of Lettuce

Wood fiber substrates with high carbon to nitrogen ratios significantly reduced the supply fast-acting nitrogen, consistent with previous findings on microbial-mediated nitrogen immobilization in organic substrates [29]. The present study showed a significant reduction in ammoniacal and nitrate nitrogen content in the wood fiber substrate compared to the control (Figure 1), which aligns with Wright et al.’s [12] findings that an additional 100 mg·L^−^^1^ nitrogen fertilizer is required for the wood substrate. Notably, despite the same total nitrogen application, lettuce biomass and total nitrogen content in the wood fiber substrate were significantly lower than in the control (Figure 2), indicating a significant inhibition of nitrogen uptake efficiency. This phenomenon may be attributed to the strong immobilization of inorganic nitrogen by microorganisms in the wood fiber substrate, converting exogenous nitrogen into organic forms and reducing effective nitrogen accumulation in plants [30]. Notably, even with 40% wood fiber in the substrate, lettuce exhibited significant nitrogen deficiency symptoms (yellowing leaves and plant dwarfism), supporting previous recommendations that wood fiber should not exceed 30% of the substrate [31]. Furthermore, nitrogen immobilization in the wood fiber substrate also affected lettuce total phosphorus and potassium content, with the gap becoming more pronounced over time. This phenomenon may be related to limited root development and down-regulation of nutrient transport protein expression due to nitrogen deficiency [32].

### 3.2. Effect of Wood Fiber Substrate on the Structure and Function of PSII and PSI in Lettuce

Chlorophyll a fluorescence transients provide a sensitive assay for assessing photosynthetic responses to abiotic stresses [26]. In this study, we revealed multiple mechanisms of damage to the lettuce photosystem caused by the wood fiber substrate, by analyzing the variable fluorescence curve (ΔV_t_) and characteristic bands (L and K bands).

Wood fiber substrates significantly affected electron transfer in both photosystem II (PSII) and photosystem I (PSI) of lettuce. Relative variable fluorescence (V_J_) at the J step reflects the efficiency of electron transfer from Q_A_^−^ to Q_B_^−^ on the PSII receptor side [24], And step I (V_I_) characterises the redox capacity of the PSI receptor side plastoquinone (PQ) library. In the present study, V_J_ and V_I_ values were significantly higher under wood fiber treatment compared to control (Figure 3), indicating that electron transfer was blocked on both PSII donor side (OEC) and PSI acceptor side. Notably, V_J_ and V_I_ values in the wood fiber group increased simultaneously at low and medium nitrogen levels, while only V_I_ values were significantly higher in other treatments. This suggests that nitrogen deficiency first affects electron transfer on the PSI acceptor side, and exacerbates damage to the PSII donor side only under severe nitrogen stress. This dose-dependent effect is consistent with Guo et al.’s study on the decline of photosystem function under nitrogen stress, which may be related to reduced ferredoxin-NADP^+^ reductase (FNR) activity and disruption of cyst-like body membrane structure caused by nitrogen deficiency [33]. At 30 days, V_J_ and V_I_ values were significantly higher only under low nitrogen treatments, with no significant difference between the wood fiber and control groups. This suggests that the negative effects of the wood fiber substrate diminished as the reproductive period progressed, but the inhibitory effects of severe low nitrogen stress on photosystem function persisted.

Structural damage on the donor side of PSII is further revealed by the kinetic features of the L and K peaks. The L peak indicates reduced connectivity of the antenna complex to reaction centers (RCs) [34,35], and the K peak reflects impaired OEC function [36]. In this study, the control group at 15 days of age showed L and K peaks only at low nitrogen levels, while the wood fiber group showed significant L and K peaks at all nitrogen levels (Figure 5), further confirming that the wood fiber substrate significantly exacerbated structural damage on the PSII donor side. At 30 days, distinct L and K peaks were observed only in the low-nitrogen wood fiber group, suggesting that the negative effects of the wood fiber substrate diminished with increased fertility, but the inhibitory effects of severe low-nitrogen stress on OEC function persisted.

A reduction in W_OI_ amplitude indicates shrinkage of the PSI-terminal electron acceptor pool (ferredoxin-NADP+ reductase) capacity [24]. Reduced nitrogen application decreased the size of the PSI receptor-side terminal electron acceptor pool, with stronger inhibition in the wood fiber group at the same nitrogen level (Figure 6). This may be related to restricted FNR synthesis due to reduced bioavailability of micronutrients (e.g., iron) in the wood fiber substrate [37]. This result, along with the 19.81–27.9% reduction in δRo in the wood fiber group (Figure 7), supports the mechanism of blocked electron transfer at the end of PSI.

Comparative analysis of chlorophyll fluorescence parameters from 15- and 30-day-old lettuce revealed a stage-specific effect of nitrogen immobilization by wood fiber substrates on photosystem function. At 15 days, the relative variable fluorescence values at the K, L, and I steps in the wood fiber group were significantly higher than those in the control group (Figure 4), indicating severe inhibition of both PSII reaction centers (particularly OEC function) and PSI receptor-side electron transfer. This is consistent with the fact that nitrogen immobilization is strongest early in planting (within 2 weeks) [30]. However, at 30 days, differences between the wood fiber and control groups were no longer significant at the K and L steps, with only the I step remaining significant, indicating superior recovery of the PSII structure compared to PSI. In performance indicator analysis, both PIabs and PItotal were significantly lower in the wood fiber group than in the control at 15 days, suggesting impaired PSII and PSI performance, particularly a significant reduction in the PSI terminal electron acceptor’s reduction capacity. By 30 days, PIabs were higher in the wood fiber group than in the control, and the gap between PI_abs_ and PI_total_ narrowed, indicating compensatory restoration of photosystem function and improved synergy between PSII and PSI as nitrogen immobilization waned. These results suggest that wood fiber substrate-induced photosystem damage is reversible, with recovery potential linked to rapid PSII repair mechanisms (e.g., D1 protein turnover) and compensatory effects of PSI cyclic electron flow [38]. This finding provides a theoretical basis for optimizing the application strategy of wood fiber substrate.

### 3.3. Effect of Wood Fiber Substrates on Photosynthesis

Nitrogen immobilization by wood fiber substrates significantly inhibited chlorophyll biosynthesis in lettuce. Chlorophyll, a key pigment of the photosynthetic system, directly influences the efficiency of light energy capture and conversion [39]. Nitrogen is essential for chlorophyll porphyrin ring synthesis and also regulates pigment accumulation by influencing chloroplast developmental gene expression [25,40,41]. The reduced nitrogen availability caused by the wood fiber substrate in this study (Figure 1) directly explains the significant decline in lettuce chlorophyll content. Additionally, potassium regulates stomatal function, activates photosynthesis-related enzymes, and facilitates assimilate transport, while phosphorus plays a direct role in assimilation and photophosphorylation [42]. Thus, nitrogen deficiency, along with potassium and phosphorus shortages, further impairs lettuce photosynthetic performance.

The inhibitory effect of wood fiber substrates on photosynthesis exhibited distinct stage-dependent characteristics (Figure 10). At the early stage (15 days), stomatal limitation was the primary factor restricting photosynthesis, indicated by a 36.2–56.8% reduction in stomatal conductance (Gs) and a 2.8–10.1% decline in intercellular CO_2_ concentration (Ci). This phase may be regulated by the ABA signaling pathway, triggered by acute nitrogen deficiency, which induces stomatal closure to minimize transpirational water loss [43]. By 30 days, the net photosynthetic rate (Pn) declined significantly (by 7.2–34.1%), even though Gs and Ci had recovered to control levels or higher, indicating a shift from stomatal to non-stomatal limitation [28]. Photosystem damage at this stage was characterized by: (1) impaired electron transfer at the PSI terminus, with δRo decreasing by 13.93–25.17% and RE0/RC decreasing by 8.79–19.74% (Figure 7, Supplementary Figure **A1**). (1) Indicating an imbalance in the ferredoxin redox cycle. (2) Reduced Rubisco activity, as evidenced by elevated Ci and persistently low Pn, indicating insufficient carboxylation efficiency. (3) Reduced photosynthetic complexes: chlorophyll content declined by 13.7–36.2%, leading to a lower density of photosystem reaction centers (RCs). This transition aligns with Cetner et al.’s theory that nitrogen deficiency triggers adaptive remodeling of photosynthetic organs, where plants prioritize basal metabolism over light energy conversion efficiency under prolonged nitrogen stress [23].

Wood fiber substrates likely inhibit photosynthesis through three primary mechanisms: (1) reduced chlorophyll synthesis, which limits light energy capture; (2) decreased stomatal conductance, leading to a lower net photosynthetic rate; and (3) structural impairment of the PSII and PSI systems. Notably, at 30 days, the difference between PIabs and PItotal narrowed in the wood fiber group, indicating a partial compensatory recovery of photosystem function. This suggests that wood fiber-induced damage was not entirely irreversible. These findings provide a theoretical basis for dynamically adjusting nitrogen supply during the reproductive period to mitigate its adverse effects.

## 4. Materials and Methods

### 4.1. Experimental Materials and Treatment Setup

The trial was conducted and completed in September 2023 at the Institute of Urban Agriculture, Chinese Academy of Agricultural Sciences. The substrate materials used in the trial included wood fiber, peat, vermiculite, and perlite. Wood fiber was sourced from Shanghai Meizijia Horticulture Co., Ltd. (Shanghai, China), whereas peat, vermiculite, and perlite were purchased from the market. Table 2 presents the substrate types: control substrate (CK) and wood fiber substrate (T). The control substrate was formulated as V_peat_:V_perlite_:V_vermiculite_ = 9:3:1. In order to exclude the interference of other factors, we screened the wood fibre substrate for group allocation ratio before the experiment to control the physicochemical properties which were basically the same as the control substrate. The final formulation was determined as V_wood fiber_:V_peat_:V_perlite_ = 2:2:1.

The second experimental factor was nitrogen level, which was adjusted based on Hoagland’s nutrient solution [44]. Three nitrogen levels were established by varying the nitrogen concentration: low nitrogen (30% N, LN), medium nitrogen (60% N, MN), and high nitrogen (100% N, HN). The concentrations of other nutrients remained essentially unchanged, and the detailed nutrient solution formulations are presented in Table A1. Six treatment groups were included in the study: high nitrogen & control(HC), high nitrogen & treatment (HT), medium nitrogen &control (MC), medium nitrogen & treatment (MT), low nitrogen & control (LC), and low nitrogen & treatment (LT), with each treatment having 10 replicates.

### 4.2. Plant Cultivation and Sampling

The test crop was cream lettuce (*Lactuca sativa cv.*) and square plastic boxes (8 cm × 8 cm × 8 cm) filled with substrate were used for the cultivation experiment. Seeds were germinated at a temperature of 22 °C and 70% humidity, shaded with a shade cloth. At 21 days after lettuce sowing, when lettuce had two leaves took the seedlings with even growth and set them in the cultivation box, one plant per box. They were placed in a light incubator with the temperature set at 22 °C for 14 h during the day and 18 °C for 10 h at night. Humidity 70%, light intensity 30,000 Lux. The light quality is white LED light. An equal amount of nutrient solution was applied to each cultivation box every 5 days during crop growth.

Analytical tests were carried out by randomly taking five plants from each treatment on the 15th and 30th day after planting, respectively. The tests consisted of three categories, namely (1) chlorophyll fluorescence curve and photosynthesis analysis of lettuce were firstly carried out, (2) Lettuce was harvested and its aboveground fresh weight, nutrient content, and chlorophyll content were determined, and (3) substrates were collected and their nitrogen fixation and ammonia and nitrate-nitrogen concentrations were determined.

### 4.3. Determination of Physico-Chemical Properties of Cultivated Substrates

#### 4.3.1. Determination of Physical and Chemical Properties of Substrates

Physical properties of the substrates, including bulk density, total porosity, air voids, and water-holding porosity, were measured using the ring knife method. Chemical properties were analyzed with a multi-parameter tester (Mettler Toledo Instruments, Shanghai, China): pH and electrical conductivity (EC) were determined in a 1:10 (*w*/*v*) extract of air-dried substrate and distilled water. Total nitrogen (TN) content was measured using a total organic carbon analyzer (Elemental XPERT-TOC/TN). For this, 0.1 g of freshly milled substrate was digested with 5 mL concentrated H_2_SO_4_ at 375 °C, followed by analysis with an inductively coupled plasma optical emission spectrometer (ICP-OES, Thermo Scientific ICAP PRO XP) [44]. Total phosphorus (TP) and total potassium (TK) were also determined by ICP-OES, while total carbon (TC) was analyzed using an elemental analyzer. Available potassium was extracted using the ammonium acetate leaching method and quantified via flame photometry. Available phosphorus was measured using the molybdenum-antimony anti-colorimetric method. The contents of cellulose, hemicellulose, and lignin were determined via the Van Soest method (a detergent fiber analysis technique).

#### 4.3.2. Determination of Substrate Nitrogen Immobilization

According to Vandecasteele’s method [18], 350 mg N·L^−1^ of KNO_3_ was added to the material and incubated at 28 °C for 4 days. The rate of nitrogen immobilization (% N) was calculated based on the difference between the mineral nitrogen content of cultured Day0 and Day4.

The steps were as follows: firstly, KNO_3_ solution with NO_3_^−^-N content of 2000 mg·L^−1^ was prepared, 400 mL of each material was taken and weighed (M1), and 70 mL of KNO_3_ solution was added to each material separately. Pure water was added to the material to adjust the moisture content to 70%, mixed thoroughly and the wet weight M2 was recorded. Place in a 200 mL acrylic box with a perforated lid and shake to level. On day 0 of incubation, the samples were left for half an hour and weighed 5.00 g. The samples were macerated with 25 mL 2 mol·L^−1^ KCL (1:5 V soil/V solution). The extract was prepared after shaking and filtration and its concentration (C1) was determined. The material was incubated at 28 °C and 70% relative humidity for 4 days, maintaining normal aeration of the cover holes. The water content was adjusted using deionised water and maintained at 70% (*w*/*w*) during incubation. On day 4 of incubation, the mineral nitrogen concentration (C2) in the material was leached using the same method as on day 0. Nitrogen immobilization rate and nitrogen immobilization were calculated from the difference between the 0-day nitrogen concentration and the 4-day nitrogen concentration. A 100% nitrogen immobilization rate means that all mineral nitrogen in the 350 mg·L^−1^ N material is fixed.

Nitrogen immobilization rate=(C1−C2)/C1∗100%

$$Nitrogen\;immobilization=\frac{140-C2*\frac{25}{5}*M2}{0.4}({mg\cdot L}^{-1})$$

#### 4.3.3. Determination of Ammonia and Nitrate Nitrogen in Substrates

The ammonia and nitrate nitrogen contents of the extracts were determined using a continuous flow analyser (SEAL Analytical, AA3, Mequon, WI, USA) to characterise the fast-acting nitrogen content of the substrate, using a 1:5 extract of fresh substrate and 2 mol·L^−1^ KCl solution (*w*/*v*).

### 4.4. Determination of Physiological Indicators in Lettuce

#### 4.4.1. Determination of Chlorophyll Content and Plant Nutrients in Lettuce

On the day of harvest for each crop, take the first leaf of lettuce that is fully expanded and photosynthetic pigments were determined according to the Lichtenthaler spectrophotometric method, including chlorophyll a, chlorophyll b [24]. Nitrogen, phosphorus and potassium were determined as previously described for nitrogen, phosphorus and potassium in planting substrates.

#### 4.4.2. Determination of Chlorophyll a Fluorescence Parameters in Lettuce

Chlorophyll fluorescence parameters were measured on the day of harvest for each crop using a FluorPen FP110 handheld chlorophyll fluorometer, and leaves were dark-adapted for 30 min prior to measurement to ensure that all PSIIs were dark-adapted. The chlorophyll a fluorescence transient was then triggered with a beam of saturated red light (~3000 μmol·m^−2^·s^−1^) with a peak wavelength of 650 nm [45].The chlorophyll a fluorescence parameters involved in this study are shown in Table A2.

#### 4.4.3. Determination of Gas Exchange Data for Lettuce

Gas exchange data were measured on the day of harvest for each crop. The first fully expanded leaf was selected from five plants with uniform growth in each treatment, and the photosynthetic rate (Pn), intercellular CO_2_ concentration (Ci), and transpiration rate (Tr) were measured using a LI-6400 portable photosynthesizer on a sunny day between 9:30 and 11:00 a.m. The results showed that the photosynthesis rate (Pn), inter-cellular CO_2_ concentration (Ci), and transpiration rate (Tr) were all measured at the same time and in the same manner as in the other treatments. Stomatal limiting values were judged according to the formula Ls = 1 − Ci/Ca, with Ca being the ambient CO_2_ concentration of Berry et al. [46].

### 4.5. Statistical Analysis

This study was analysed by one-way ANOVA using IBM SPSS Statistics 27, significant differences between treatment means were detected using Duncan’s multiple range test (*p* < 0.05) and graphing was done using Origin 2022.

## 5. Conclusions

This study systematically examined the physiological mechanisms by which wood fiber substrates influence lettuce photosynthesis via nitrogen immobilization. The high carbon-to-nitrogen ratio of the wood fiber substrate significantly reduced the supply of fast-acting nitrogen (Figure 1), decreasing lettuce nitrogen uptake efficiency (Figure 3), triggering typical nitrogen deficiency symptoms (Figure 2), and reducing chlorophyll content (Figure 9). Chlorophyll fluorescence kinetics analysis suggests that wood fiber substrates impair photosystem function through multiple pathways: In the early stage (15 days), structural damage occurred on the donor side (OEC) of PSII, and electron transfer was blocked on the acceptor side of PSI (Figure 5). In the later stage (30 days), PSII function was partially restored, but PSI damage persisted, indicating that nitrogen deficiency caused by wood fiber nitrogen immobilization affects the photosystem in stages (Figure 6). Notably, some of the photosystem damage induced by wood fibers appears reversible. The results provide a theoretical basis for optimizing nitrogen management in wood fiber substrates and suggest that negative effects can be mitigated by dynamically regulating nitrogen supply during the reproductive period and supplementing trace elements (e.g., iron and magnesium), promoting the efficient use of wood fiber substrates in horticulture.

## Figures and Tables

**Table 1:** Nitrogen immobilization potential of wood fibers. High nitrogen (100% N, HN), medium nitrogen (60% N, MN), and low nitrogen (30% N, LN).

Form	HN	MN	LN
Nutrient solution nitrogen concentration (mg N·L^−1^)	210.30	127.60	62.50
Nutrient solution input per pot (L)	0.32	0.32	0.32
Total nitrogen input to single pot (mg)	67.30	40.32	20.00
Wood fiber nitrogen immobilization capacity (mg N·L^−1^)	115.00		
Volume of wood fiber in single pot substrate (L)	0.16		
4-day nitrogen immobilization potential of single-pot substrates (mg)	18.40		

**Table 2:** Basic physico-chemical properties of test substrates.

	Capacity (g·cm^−3^)	Porosity (%)			pH	EC (ms·cm^−1^)	Nutrients (g·kg^−1^ Dry Weight)			
		Total Porosity	Water-Holding Porosity	Air Porosity			Quick-Acting Nitrogen	Total Nitrogen	Total Phosphorus	Total Potassium
Control substrate	0.21	0.72	0.68	0.04	6.26	0.46	0.30	0.82	0.01	2.2
Wood fiber	0.18	0.8	0.72	0.07	6.09	0.42	0.24	0.65	0	1.67

## Data Availability

Data is contained within the article.

## Abbreviations

The following abbreviations are used in this manuscript:

N	Nitrogen
PSII	Photosystem system II
PSI	Photosystem system I
OEC	Oxygen evolving complex
Pn	Photosynthesis rate
Ci	Inter-cellular CO_2_ concentration
Tr	Transpiration rate
Gs	Stomatal conductance
Ls	Stomatal limiting values
CK	Control substrate
T	Wood fiber substrate
L	Low nitrogen
M	Medium nitrogen
H	High nitrogen

## Appendix A

**Table A1:** Nutrient solution formulations used in this study.

Formulation Name		Elemental (mg·L^−1^)				Nitrogen Level	Concentration of Each Element (mg·L^−1^)				
		Ca(NO_3_)_2_·4H_2_O	KNO_3_	NH_4_H_2_PO_4_	MgSO_4_·7H_2_O		N	P	K	Ca	Mg
Hogeland and Anon Universal Nutrient Solution (modified)	HN	945	607	115	493	100%	210.3	31.0	234.4	160.2	48.1
	MN	240	607	115	493	60%	126.6	31.0	234.4	40.7	48.1
	LN	0	350	115	493	30%	62.5	31.0	135.1	0.0	48.1

**Table A2:** The chlorophyll fluorescence parameters involved in this study.

Terms and Formulas	Illustrations
F_O_ = F (20 μs)	F_O_ (Minimum fluorescence): the fluorescence value in the dark-adapted state when all reaction centres are open, i.e., when Q_A_^−^ , the primary electron acceptor of the reaction centre, is in the oxidation state
F_L_ = F (150 μs)	Fluorescence value at 150 μs
F_K_ = F (300 μs)	Fluorescence value at 300 μs
F_J_ = F (3 ms)	Fluorescence value at 3 ms (J level of OJIP)
F_I_ = F (30 ms)	Fluorescence value at 30 ms (I level of OJIP)
F_M_ = F_P_ = F (t)_max_	F_M_ (maximum fluorescence): the fluorescence value when all reaction centres are switched off in the dark-adapted state
F_v_ = F_M_ − F_O_	Maximum variable fluorescence
V_L_ = (F_L_ − F_O_)/ (F_M_ − F_O_)	Relative variable fluorescence in the L phase of the fluorescence induction curve
V_K_ = (F_K_ − F_O_)/ (F_M_ − F_O_)	Relative variable fluorescence in the K phase of the fluorescence induction curve
V_J_ = (F_J_ − F_O_)/ (F_M_ − F_O_)	Relative variable fluorescence in the J phase of the fluorescence induction curve
V_I_ = (F_I_ − F_O_)/ (F_M_ − F_O_)	Relative variable fluorescence in stage I of the fluorescence induction curve
M_0_ = (ΔV/Δt)_0_ = 4 (F_300μs_ − F_O_)/ (F_M_ − F_O_) = TR_0_/RC − ET_0_/RC	Approximate initial slope (per millisecond) of transient fluorescence V = f(t).This parameter reflects the rate of closure of PS II active reaction centres (RCs).
ABS/RC = [4 (F_300μs_ − F_O_)/ (F_2ms_ − F_O_)] (F_M_/F_V_) = M_0_ (1/V_J_) (1/ϕ_Po_)	Energy flux absorbed per unit PS II active reaction centre
TR_0_/RC = ϕ _ Po _ (ABS/RC)	Energy flux captured per unit PS II active reaction centre at t = 0
ET_0_/RC = ϕ_Eo_ (ABS/RC)	Energy flux transferred per unit PS II active reaction centre at t = 0
RE_0_/RC = ϕ_Ro_ (ABS/RC) = (1 − V_I_)× (M_0_/V_J_)	Specific electron flux per unit of PS II active reaction centre reducing PS I terminal electron acceptor at t = 0
DI_0_/RC = (ABS/RC) − (TR_0_/RC)	Energy flux per unit PS II active reaction centre heat dissipation at t = 0
ϕ_Po_ = TR_0_/ABS = F_V_/F_M_ = 1 − F_O_/F_M_	The maximum quantum yield of the primary photochemical reaction at t = 0 refers to the quantum yield of photons captured by the PSII reaction centre and also indicates the maximum photochemical efficiency of the PSII reaction centre in the dark-adapted state
ψ_Eo_ = ET_0_/TR_0_ = (1 − V_J_)	Ratio of electrons captured by the active reaction centre of PS II being transferred at t = 0
ϕ_Eo_ = ET_0_/ABS = (1 − F_O_/F_M_) ψ_Eo_ = ϕ_Po_ψ_Eo_	Quantum efficiency of electron transfer from Q_A_^−^ to electron transfer chain at t = 0
ψ_Ro_ = RE_0_/TR_0_ = 1 − V_I_	Ratio of electrons captured by the active reaction centre of PSII being transferred to PSI at t = 0
δ _ Ro _ = RE_0_/ET_0_ = (1 − V_I_)/ (1 − V_J_)	Ratio of electrons in the reduced PSI receptor measuring terminal electron acceptor to electrons in the electron transport chain
ϕ_Ro_ = RE_0_/ABS = ϕ_Po_ψ_Ro_ = ϕ_Po_ψ_Eo_δ_Ro_	Quantum yields of electron acceptors at the lateral end of reduced PSI receptors
ϕ_Do_ = F_O_/F_M_ = 1 − ϕ_Po_	Thermal dissipation quantum yield at t = 0, reflecting the proportion of absorbed light energy dissipated by non-photochemical quenching processes in PSII
